# Effect of a gamified mobile‐based self‐management application on disease activity index, quality of life, and mental health in adults with inflammatory bowel disease: A protocol of a randomized controlled trial study

**DOI:** 10.1002/hsr2.2109

**Published:** 2024-05-21

**Authors:** Narges Norouzkhani, Mahbobeh Faramarzi, Ali Bahari, Javad Shokri Shirvani, Yeganeh Ebrahimnia Shirvani, Saeid Eslami, Hamed Tabesh

**Affiliations:** ^1^ Department of Medical Informatics, Faculty of Medicine Mashhad University of Medical Sciences Mashhad Iran; ^2^ Population, Family and Spiritual Health Research Center, Health Research Institute Babol University of Medical Sciences Babol Iran; ^3^ Department of Internal Medicine, Faculty of Medicine Mashhad University of Medical Sciences Mashhad Iran; ^4^ Department of Internal Medicine Babol University of Medical Sciences Babol Iran; ^5^ Student Research Committee, School of Medicine Shahroud University of Medical Sciences Shahroud Iran; ^6^ Pharmaceutical Research Center Mashhad University of Medical Sciences Mashhad Iran

**Keywords:** applications, inflammatory bowel diseases, mental health, quality of life, self‐management

## Abstract

**Background and Aims:**

Inflammatory bowel disease (IBD) is a chronic inflammatory gastrointestinal tract disease subdivided into Crohn's disease (CD) and ulcerative colitis (UC). There is currently no cure for IBD, and individuals with IBD frequently experience a lower health‐related quality of life (HRQOL) than the general population. Gamification has become an increasingly popular topic in recent years. Adapting game design concepts to nongaming contexts represents a novel and potential approach to changing user engagement. This study will be conducted with the aim of evaluating the effect of a gamified mobile‐based self‐management application on disease activity index, quality of life, and mental health in adults with IBD.

**Methods:**

A multicenter, parallel, two‐arm, exploratory randomized controlled trial with a 6‐month follow‐up per patient will be designed to compare the impact of the gamified mobile‐based tele‐management system on primary and secondary health outcomes and outpatient visits in 210 patients with all types of IBD which are divided equally into a control group with standard care and an intervention group which will use the developed mobile application named *MY IBD BUDDY*. All patients will attend study visits at baseline, 12 and 24 weeks, and routine IBD clinic visits or telephone consultations based on randomization group assignment. Disease activity or disease activity index, mental health (anxiety and depression) symptoms, quality of life, self‐efficacy, and IBD‐specific knowledge will be measured at baseline with two follow‐ups at 12 and 24 weeks.

**Conclusions:**

In sum, the outcomes of our trial will demonstrate the impact of the gamified mobile‐based self‐management system on disease activity, quality of life, and anxiety and depression by means of interactive care and patient empowerment.

**Trial Registration:**

IRCT: IRCT20200613047757N1. Registered November 16, 2021. Prospectively registered and visible at OSF (https://doi.org/10.17605/OSF.IO/AWFY9).

## 1 INTRODUCTION

### 1.1 Background

Inflammatory bowel disease (IBD) is a chronic inflammatory gastrointestinal tract disease subdivided into Crohn's disease (CD) and ulcerative colitis (UC).^1^ It is caused by an exaggerated immune system reaction to a normal stimulus, such as food and intestinal flora, in genetically susceptible individuals.^2^ In North America, UC has 2.2–19.2 instances per 100,000 person‐years, while CD has 3.1–20.2 per 200,000. An extensive insurance claims analysis found 238 and 201 cases of adult UC per 100,000 Americans. North America and Europe have more IBD than Asia or Africa. Up to 25% of individuals acquire IBD by adolescence. A second 10%–15% peak developing IBD beyond 60 suggests a bimodal distribution. According to a considerable investigation based on insurance claims, the prevalence of UC in adults in the United States was 238 per 100,000 and 201 per 100,000. IBD is more prevalent in North America and Europe than Asia and Africa.^3^

### 1.2 IBD self‐management

Self‐management is an interactive and evolving process that captures the complexities of coping with chronic illness within the context of daily life.^4^ It involves acquiring skills that help individuals effectively manage their chronic conditions on a day‐to‐day basis. self‐management involves three main tasks^5^: dealing with illness‐related challenges, addressing everyday life responsibilities, and reflecting on one's life experiences. Building upon this definition,^6^ identified five essential self‐management skills: problem‐solving, decision‐making, resource utilization, establishing partnerships with healthcare professionals, and taking proactive steps to manage one's health. There is currently no cure for IBD, and individuals with IBD frequently experience a lower health‐related quality of life (HRQOL) than the general population.^7^ An increasing corpus of research indicates that effective self‐management contributes to enhanced health outcomes, including pain, disability, and healthcare utilization, in various populations. However, limited is known about the different parts and effects of self‐management among IBD patients.^8^

### 1.3 Gamification conceptual

Gamification has become an increasingly popular topic in recent years. Adapting game design concepts to nongaming contexts represents a novel and potential approach to changing user engagement.^9^ Gamification design benefits numerous fields, including e‐commerce, education, and healthcare.^10^ Combining psychology and technology to achieve business objectives, however, the function of technology in gamification has evolved significantly. Mobile health is the next suitable surge of technologies for modulating patient involvement and activities, reshaping gamification design in health care due to its unique technological capabilities.^11^ Combining the concept of a gamified mobile‐based self‐management system with IBD could result in the development of a mobile application designed to aid individuals with IBD in more effectively managing their condition.^12^

The present platform could be used in various fields such as Social Support Community Engagement, Symptom Management, Goal Setting and Progress, Education and Resources, and Tracking or Reminders.^13^ By gamifying the self‐management manipulation, IBD individuals may feel inspired, involved, and empowered to take an active role in their health management, resulting in improved symptom control, and improved health.^14^ To our knowledge, this research is the first study that has focused on the design of self‐care and monitoring systems for IBD patients with gamification techniques.

This study is a preliminary research to provide an explanation of the research protocol, offering a step‐by‐step approach and its methodology, and in the second paper, the results will be analyzed and reported.

## 2 METHODS

### 2.1 Objective and aims

**Figure 1:**
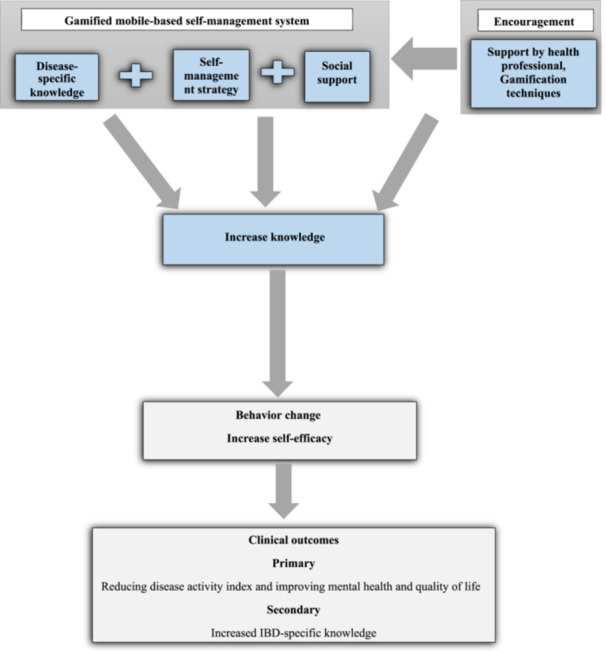
Conceptual model outlining the hypothesized mechanism of action of the IBD adult patients charge: managing IBD gamified mobile‐based intervention. IBD, inflammatory bowel disease.

The main goal of this study is to evaluate the efficacy of a gamified mobile‐based self‐management intervention system in reducing disease activity index and improving mental health and quality of life in adults with IBD compared to standard routine care. The authors hypothesize that the use of this new intervention system will result in lower disease activity index, improved anxiety and depression symptoms, improved quality of life, increased self‐efficacy, and IBD‐specific knowledge. The hypothesized mechanism of action of the self‐management intervention on health outcomes is summarized in Figure 1. This conceptual model is based on Murray et al.'s research.^15^

### 2.2 Study setting

Adult IBD patients will be recruited in person from three Iranian adult gastroenterology and Hepatology centers. A local research coordinator will explain the study to eligible patients and obtained written consent.

### 2.3 Eligibility criteria

#### 2.3.1 Inclusion criteria

(1) Participants must have been diagnosed with IBD according to internationally accepted criteria at least 6 months before the start of the study, (2) age ≥ 18 years, (3) able to speak and read Persian (Farsi), (4) willing and able to provide written consent, and (5) familiarity with and access to an android smartphone or tablet, and internet.

#### 2.3.2 Exclusion criteria

Patients meeting the following criteria are excluded from the study:

- Inability to communicate with the research team and adherence to study requirements.
- Unwillingness to sustain participation.
- Pregnancy.
- Active perianal disease or anal fistula.
- Ileostomy, colostomy, pouch anastomosis, or ileorectal anastomosis.
- Pending or imminent surgery.
- Participation in another experimental study or educational interventions throughout patient enrollment.
- Uncontrolled and significant comorbidities or cognitive impairments.
- Patients with a hospital admission within 2 weeks before the study will be excluded for ethical reasons.

### 2.4 Study procedure and design

In the present study, a gamified mobile‐based tele‐management system for patients with all types of IBD will be developed. To compare the impact of the gamified mobile‐based tele‐management system on health outcomes and outpatient visits in patients with all types of IBD, the authors will design a multicenter, parallel, two‐arm, exploratory randomized controlled trial with a 6‐month follow‐up per patient. IBD patients will be recruited from general practices and outpatient clinics after completing baseline questionnaires and providing informed consent. Subsequently, eligible patients will be randomly assigned to either (A) self‐management support through the use of the gamified mobile‐based tele‐management system (intervention group, MyIBD Buddy app), or (B) standard IBD care (control group). All patients will attend study visits at baseline, 12 and 24 weeks, and routine IBD clinic visits or telephone consultations based on randomization group assignment. Disease activity, anxiety and depression symptoms, quality of life, self‐efficacy, and IBD‐specific knowledge will be measured at baseline and during the 24‐week follow‐up. The Harvey–Bradshaw index (HBI) will measure disease activity among CD patients. Also, the disease activity among UC patients will be investigated using the simple clinical colitis activity index (SCCAI, also known as the Walmsley index). Notably, the Research Ethics Committee of Mashhad Faculty of Medical Sciences approved the current study in November 2021 (IR.MUMS.REC.1400.230). The trial prospectively registered is at IRCT (IRCT20200613047757N1) and visible at OSF (https://doi.org/10.17605/OSF.IO/AWFY9).

### 2.5 Sample size

With a significance level of 5%, statistical power of 80%, a correlation coefficient of at least 0.3 between the scales, and three measurements of the results—once at baseline and twice after randomization—repeated measures analysis will be used to calculate the sample size. The results of relevant publications will be used as the foundation for the analysis of covariance (ANCOVA) for various indicators. The sample size was calculated using the Sampsi module and the minimum clinically important difference (MCID) effect technique in the Stata 2015 environment. Consequently and based on the enormous sample size available for the quality of life index and accounting for the likelihood of a 20% dropout for each of the three measurements of the outcome, the sample size for this research is 105 participants in each group and 210 participants will be recruited.

### 2.6 Random assignment

Patients will be randomly assigned to the intervention or control group using a stratified block randomization sequence generated by WINPEPI, with the individual as the randomization unit. All IBD patients from the participating centers will be invited to participate in this investigation. An independent administrator will generate the allocation sequence and prepare 210 numbered, opaque, sealed envelopes containing assignments distributed equally between the two study groups. Each participant will be notified via email or the social network of their designated group and given instructions on how to access the relevant app or online survey.

### 2.7 Study groups

After obtaining informed consent, the patients will be stratified by disease type, disease activity, and location of outpatient appointments. The participants will then be assigned to the intervention (A) or control (B) group according to the procedure described in 2.7. The groups are described in the following sections.

#### 2.7.1 Group A or intervention (gamified mobile‐based tele‐management system)

Participants in the gamified mobile‐based tele‐management system (MyIBD Buddy application) group will receive instructions on using the system and a username and password which will be valid for up to 6 months via standard care. The application was developed for the Android platform using the Suker Wheel game development framework. The research team closely collaborated with the development team and the five‐step ADDIE design model was utilized. The goal was to combine behavior modification strategies and self‐management theory. The educational content of this application has been studied and validated by the research team in our previous work.^16^, ^17^, ^18^, ^19^, ^36^ The study outlines the development process of an application aimed at teaching self‐care techniques, including self‐care skills and psychological aspects such as stress and anxiety control, to monitor patients' physical and mental status, including self‐care skills and psychological aspects such as stress and anxiety control, to monitor patients' physical and mental status, relaxation and mindfulness techniques.

**Figure 2:**
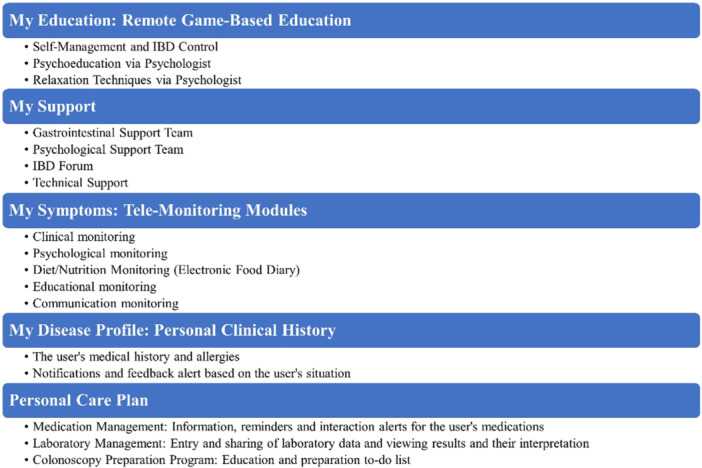
The main functionalities offered by the system. IBD, inflammatory bowel disease.

**Figure 3:**
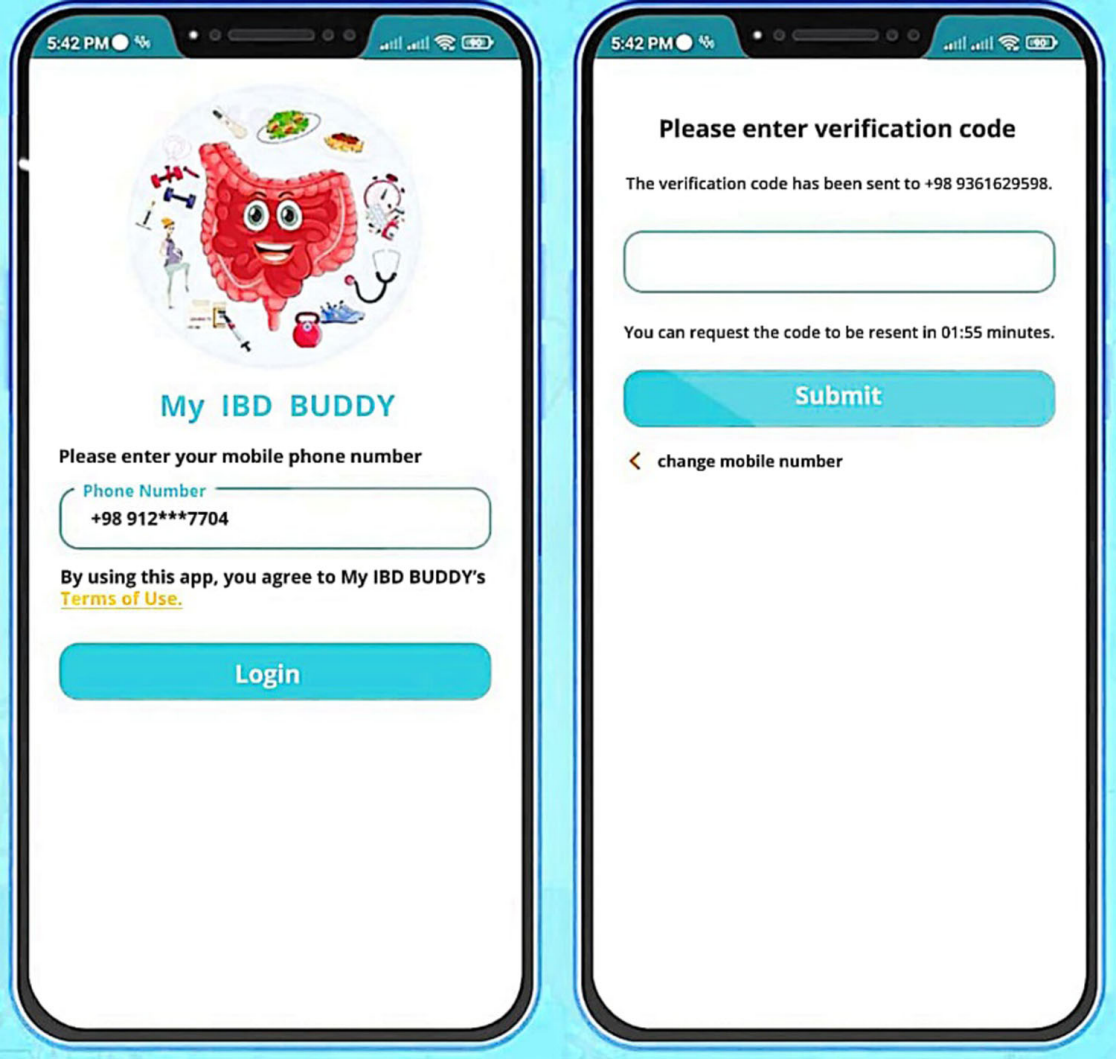
Sign‐up of the “MY IBD BUDDY.”

**Figure 4:**
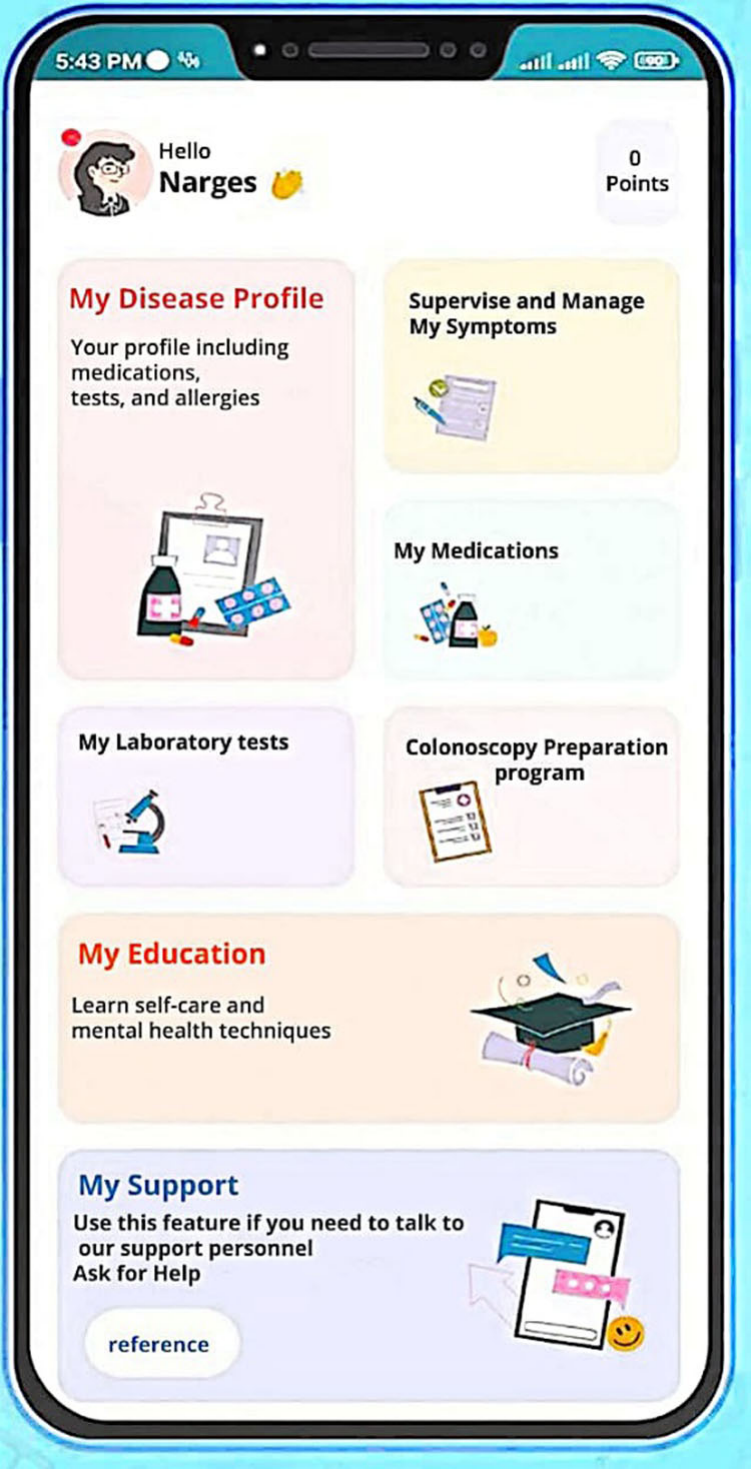
Home page of the “MY IBD BUDDY.”

The main functionalities of the MY IBD Buddy application include the following components (Figures 2, 3, 4):

- Login page includes user information and disease registration.
- Patient disease profile (clinical disease history, body mass index calculator).
- Tele‐monitoring, including five components (tracking symptoms related to IBD):

1. Interaction monitoring (assessing satisfaction with the application).
2. Psychological and mental health monitoring (monitoring anxiety and depression, IBD quality of life, and IBD self‐efficacy).
3. Medical monitoring (disease activity using disease activity and providing feedback, fecal calprotectin levels monitoring).
4. Educational monitoring (assessing knowledge related to the IBD).
5. Nutritional monitoring (food diary).

- Medication management (medication registration, viewing medication information, setting medication reminders, verifying IBD‐medication interactions, approved and unapproved medication for IBD patients, authorized and unauthorized medication during pregnancy and breastfeeding for patients with IBDs).
- Laboratory management (manual and visual entry of tests).
- Colonoscopy readiness program (list and electronic form of precolonoscopy tasks along with setting reminders, comprehensive pre‐ and postcolonoscopy instructions).
- Tele‐education (self‐care education for managing IBD, and psychological education).
- Support module (clinical engagement with gastroenterology and mental health experts, technical support for the app, and discussion forum for individuals with IBD).

In this application, there are various challenges and tasks for users to earn points. These points are awarded based on the user's progress displayed through the leaderboard and points table, level‐up, and progress bar to serve as an incentive for other users and to create competition. However, this competition is not related to caregiving tasks or medical test results, but rather to the number of interactions the user has with the system and, in fact, to the user experience within the system. For example, each user earns points by studying a chapter of the tutorial, doing gamifying tests, leveling up, and unlocking the next tutorial chapters. Additionally, by completing monitoring forms related to disease symptoms, medication registration, medication alert, test registration, and participating in educational challenges, the user earns points.

#### 2.7.2 Group B (standard care)

The individuals in the conventional care group will receive IBD treatment from their gastroenterologist. The standard of care is based on current evidence‐based professional guidelines, which include a comprehensive assessment, a guideline‐concordant therapy plan, scheduled and as‐needed clinic visits, scheduled and as‐needed telephone calls, and the administration of educational fact sheets about disease‐specific topics as appropriate. They will be instructed to complete questionnaires online or by hand and will receive three complimentary visits to maintain their participation in the study. Participants in Group B will be given access to the gamified mobile‐based tele‐management program after 6 months of data collection.

### 2.8 Masking

Participants and the program coordinator cannot be blinded to participant study allocation due to the nature of the intervention. However, the statistician who will validate the data analysis will be blinded to the randomization groups.

### 2.9 Data collection and study outcomes

Table 1 shows the data extraction procedure.

**Table 1:** The procedure of data collecting.

Measure	Measurement method
Primary outcomes	
Quality of life	HRQOL will be measured using the IBDQ^20^ The IBDQ contains 32 items divided into four health subdimensions: bowel symptoms, systemic symptoms, social functioning, and emotional function. The total score ranges from 32 to 224, and a higher score indicates a better quality of life.^20^, ^21^ A clinically significant change score is +16.^22^, ^23^, ^24^, ^25^
Disease activity scores	Disease activity scores are calculated using the HBI^26^ for participants with CD and the SCCAI^27^ for participants with UC/indeterminate colitis. Patients with CD and an HBI < 5 will be considered to be in clinical remission, whereas patients with scores of 5–7, 8–16, or >16 will be classed, respectively, as having mild, moderate, or severe activity.^26^ For patients with UC, clinical remission will be defined as an SCCAI ≤ 2, whereas values of 3–5 or >5 points will be classed as mild‐to‐moderate or severe activity.^28^
Anxiety and depression	Hospital Anxiety and Depression Scale (HADS): The 14 items on the HADS are equally divided between the HADS‐Anxiety and HADS‐Depression subscales. Subscale scores of 8–10 are categorized as borderline and 11–21 as clinical.^29^, ^30^ A clinically significant change score is −1.5 to −2 points.^31^, ^32^
Secondary outcomes	
Self‐efficacy	Self‐efficacy will be measured with the 29‐item inflammatory bowel disease self‐efficacy scale (IBD‐SES)^33^ Questions are grouped into four domains: managing stress and emotions, managing medical care, managing symptoms and disease, and maintaining remission. Overall scores on the IBD‐SES range from 29 to 290, with higher scores indicating higher self‐efficacy and thus better self‐management and greater patient empowerment.
Patient knowledge	As assessed by the IBD‐KNOW, patient knowledge will be compared between groups. The total score ranges from 1 to 24, and a higher score indicates a higher disease knowledge.^34^
Patient satisfaction (user experience)	Using a 5‐point figurative scale from 1 (*very satisfied*) to 5 (*not satisfied*).

*Note*: Follow‐ups will be done at 0, 12, and 24 weeks after randomization except for the last item.

Abbreviations: CD, Crohn's disease; HRQOL, health‐related quality‐of‐life; IBDQ, inflammatory bowel diseases questionnaire; SCCAI, simple clinical colitis activity index; UC, ulcerative colitis.

### 2.10 Data management

First, the characteristics of the patients in the test and control groups will be documented, and any differences between the groups will be assessed using tests to measure variations in means or proportions. Second, the intergroup variations in disease outcomes will be assessed using multivariable linear regression which will be adjusted for the following stratification criteria: subtypes of IBD (CD or UC), disease activity at baseline (remission or active), age (numerical), sex (male or female), disease duration (numerical), smoking (nonsmoker, active smoker, or ex‐smoker), and educational level (five levels). For missing outcomes and variables, the multiple imputation approach will be used to impute the missing data. Results will be adjusted for age, sex, and illness duration, disease activity at baseline, educational attainment, and stratification criteria.

### 2.11 Statistical analysis

These models' adjusted intervention effects and 95% confidence interval and *p* values will be provided. Statistical significance will be determined by a two‐sided *p* value > 0.05. Statistical analysis will be conducted to understand the characteristics of patients with missing data.

Using SPSS's multiple imputation approach, missing data at the case level will be imputed. For the statistical analysis, SPSS V.22 will be used.

#### 2.11.1 Primary study analysis

The initial outcome analyses investigated mean disease activity measures within disease type at baseline and each of the measurement; repeated measures analysis of variance (ANOVA) will be used to compare the intervention groups and standard care at these time points. Also, repeated measures ANOVA will be used to compare each group's change from baseline at 12 and 24 weeks, within disease type. The two groups will be combined in the same analyses if a low variation is identified. Plots of mean outcome values in each group will be examined for indications of change patterns. Similar analyses will be used for quality of life and anxiety and depression scores by groups and for all participants.

Furthermore, the primary goal of these repeated measures analyses will be to detect differences between the intervention groups in patterns of change from baseline across the 6 months. Regression diagnostics will be examined to detect outliers, influential observations, and other deviations from assumptions for all models. To detect potential bias by analysis of only the collected data, participants with and without data at a given time will be compared to determine the baseline characteristics of those who did not provide complete data.

#### 2.11.2 Secondary outcomes analysis

The secondary outcome will be analyzed using the previous approach. Linear regression models that account for potential association‐confounding factors will be established. In a secondary analysis, repeated measures models will examine recurrence and remission patterns according to arm type.

## 3 DISCUSSION

This protocol describes a randomized controlled trial (RCT) to test and evaluate the efficacy of a gamified mobile‐based self‐management system intervention in reducing disease activity index and enhancing mental health and quality of life in adults with IBD compared to a control condition of standard routine care. The present investigation will examine the self‐management intervention's further hypothesized mechanism of action on the outcomes of IBD health management.

### 3.1 Strengths and limitations

The current protocol has two critical strengths. First, it will be the first IBD study to characterize both IBD's psychological and somatic aspects with gamification techniques. Second, this research has the potential to enhance patients' self‐care abilities.

Thirdly, the treatment will consist of remote mobile game‐based multimodal cognitive training to lengthen the sessions while maximizing adherence.^35^ Lastly, this will be one of the few studies on mobile applications to examine long‐term outcomes following training, providing information on the neuropsychological and somatic effects on IBD patients over the long term.

This protocol has a limitation as well. One of the inherent limitations of such studies, self‐report assessment, is reporting bias. However, high response rate will attenuate this weakness and promote the notion that the sample population is a good representative of the overall IBD patients. Also, generalizability of the findings may be restrained by unique characteristics of the population. Needs, preferences, and beliefs of one population are not similar to other peers from other countries, societies, and cultures. Future investigations can be extended to other populations. This study compared the impact of standard treatment to the effect of adding a home‐based mobile multimodal cognitive training program to determine if this additional treatment is beneficial.

Totally, if online platforms and home‐based IBD education are effective, it can be a cost‐effective intervention for people with this disease and their families, with regular and cost‐effective surveillance of these people throughout the disease's progression, which can also enhance the quality of life.

## 4 CONCLUSIONS

Our primary aim was to assist other researchers in developing tele‐management interventions, selecting appropriate methods for remotely measuring health outcomes, and identifying patient groups that would benefit from this approach in future IBD studies. The outcomes of our trial will demonstrate the impact of the gamified mobile‐based self‐management system on disease activity, quality of life, and anxiety and depression by means of interactive care and patient empowerment. It is imperative to undertake additional studies to authenticate the effectiveness of the *MY IBD BUDDY* tele‐management program in other specific patient groups, such as those with uncomplicated diseases, limited access to medical care or social support, and patients who cannot attend specialized IBD clinics.

## AUTHOR CONTRIBUTIONS

**Narges Norouzkhani**: Conceptualization; investigation; writing—original draft; software; visualization. **Mahbobeh Faramarzi**: Investigation; methodology; writing—review and editing. **Ali Bahari**: Writing—review and editing; data curation; resources. **Javad Shokri Shirvani**: Writing—review and editing; data curation. **Yeganeh Ebrahimnia Shirvani**: Data curation; writing—review and editing. **Saeid Eslami**: Investigation; writing—review and editing; methodology. **Hamed Tabesh**: Writing—review and editing; conceptualization; investigation; methodology; validation; formal analysis; project administration; supervision.

## CONFLICT OF INTEREST STATEMENT

The authors declare no conflict of interest.

## ETHICS STATEMENT

The study was conducted according to the guidelines of the Declaration of Helsinki, and approved by the Ethics Committee of Mashhad Faculty of Medical Sciences in November 2021 (protocol code: IR.MUMS.REC.1400.230 and date of approval: November 2021).

## TRANSPARENCY STATEMENT

The lead author Hamed Tabesh affirms that this manuscript is an honest, accurate, and transparent account of the study being reported; that no important aspects of the study have been omitted; and that any discrepancies from the study as planned (and, if relevant, registered) have been explained.

## Data Availability

The original data presented in the study are included in the article; the data supporting the results of the present study are only available from the authors upon reasonable request and with permission of [Hamed Tabesh and Narges Norouzkhani].

## References

[1] Maaser C , Sturm A , Vavricka SR , et al. ECCO‐ESGAR guideline for diagnostic assessment in IBD part 1: initial diagnosis, monitoring of known IBD, detection of complications. J Crohn's Colitis. 2019;13(2):144‐164. 30137275 10.1093/ecco-jcc/jjy113

[2] Dmochowska N , Wardill H , Hughes P . Advances in imaging specific mediators of inflammatory bowel disease. Int J Mol Sci. 2018;19(9):2471. 30134572 10.3390/ijms19092471 PMC6164364

[3] Su HJ , Chiu YT , Chiu CT , et al. Inflammatory bowel disease and its treatment in 2018: global and Taiwanese status updates. J Formos Med Assoc. 2019;118(7):1083‐1092. 30054112 10.1016/j.jfma.2018.07.005

[4] Conley S , Redeker N . A systematic review of self‐management interventions for inflammatory bowel disease. J Nurs Scholarsh. 2016;48(2):118‐127. 26756193 10.1111/jnu.12189 PMC5480612

[5] Corbin JM , Strauss A . Unending Work and Care: Managing Chronic Illness at Home. Jossey‐Bass; 1988.

[6] Lorig KR , Holman HR . Self‐management education: history, definition, outcomes, and mechanisms. Ann Behav Med. 2003;26(1):1‐7. 12867348 10.1207/S15324796ABM2601_01

[7] Bernklev T , Jahnsen J , Lygren I , Henriksen M , Vatn M , Moum B . Health‐related quality of life in patients with inflammatory bowel disease measured with the short form‐36: psychometric assessments and a comparison with general population norms. Inflamm Bowel Dis. 2005;11(10):909‐918. 16189421 10.1097/01.mib.0000179467.01748.99

[8] Barlow J , Wright C , Sheasby J , Turner A , Hainsworth J . Self‐management approaches for people with chronic conditions: a review. Patient Educ Couns. 2002;48(2):177‐187. 12401421 10.1016/s0738-3991(02)00032-0

[9] Deterding S, Sicart M, Nacke L, O'Hara K, Dixon D. Gamification Using Game‐Design Elements in Non‐Gaming Contexts: *CHI'11 Extended Abstracts on Human Factors in Computing Systems*. ACM; 2011:2425‐2428.

[10] Zichermann G , Cunningham C . Gamification by Design: Implementing Game Mechanics in Web and Mobile Apps. O'Reilly Media, Inc; 2011.

[11] Nguyen HD, Jiang Y, Eiring Ø, Poo DCC, Wang W, eds. Gamification Design Framework for Mobile Health: Designing a Home‐Based Self‐Management Programme for Patients With Chronic Heart Failure. Paper presented at: *Social Computing and Social Media Technologies and Analytics: 10th International Conference, SCSM 2018*; Held as Part of HCI International 2018; July 15–20, 2018; Las Vegas, NV; Proceedings, Part II 10; 2018: Springer.

[12] Lalloo C , Jibb LA , Rivera J , Agarwal A , Stinson JN . There's a Pain App for that. Clin J Pain. 2015;31(6):557‐563. 25370138 10.1097/AJP.0000000000000171

[13] Naslund JA , Aschbrenner KA , Araya R , et al. Digital technology for treating and preventing mental disorders in low‐income and middle‐income countries: a narrative review of the literature. Lancet Psychiatry. 2017;4(6):486‐500. 28433615 10.1016/S2215-0366(17)30096-2 PMC5523650

[14] Plevinsky J , Greenley R , Fishman L . Self‐management in patients with inflammatory bowel disease: strategies, outcomes, and integration into clinical care. Clin Exp Gastroenterol. 2016;9:259‐267. 27601930 10.2147/CEG.S106302 PMC5003515

[15] Murray E , Burns J , See TS , Lai R , Nazareth I . Interactive health communication applications for people with chronic disease. Cochrane Database Syst Rev. 2005;4:CD004274. 10.1002/14651858.CD004274.pub4 PMC13184810 16235356

[16] Norouzkhani N , Bahari A , Faramarzi M , Shokri Shirvani J , Eslami S , Tabesh H . Development and validation of an educational book on self‐management in inflammatory bowel disease based on patient preferences and expert opinions: a methodological study. J Clin Med. 2023;12(24):7659. 38137727 10.3390/jcm12247659 PMC10744084

[17] Norouzkhani N , Bahari A , Shirvani JS , Faramarzi M , Eslami S , Tabesh H . Expert opinions on informational and supportive needs and sources of obtaining information in patients with inflammatory bowel disease: a Delphi consensus study. Front Psychol. 2023;14:1224279. 37809295 10.3389/fpsyg.2023.1224279 PMC10557489

[18] Norouzkhani N , Faramarzi M , Ghodousi Moghadam S , et al. Identification of the informational and supportive needs of patients diagnosed with inflammatory bowel disease: a scoping review. Front Psychol. 2023;14:1055449. 37251032 10.3389/fpsyg.2023.1055449 PMC10211349

[19] Norouzkhani N, Tabesh H, Moghadam SG. *Identifying the Informational and Supportive Needs of Patients Diagnosed With Inflammatory Bowel Disease: A Scoping Review*. OSF; 2022. 10.3389/fpsyg.2023.1055449 PMC10211349 37251032

[20] Irvine EJ , Feagan B , Rochon J , et al. Quality of life: a valid and reliable measure of therapeutic efficacy in the treatment of inflammatory bowel disease. Gastroenterology. 1994;106(2):287‐296. 8299896 10.1016/0016-5085(94)90585-1

[21] Guyatt G , Mitchell A , Irvine EJ , et al. A new measure of health status for clinical trials in inflammatory bowel disease. Gastroenterology. 1989;96(2):804‐810. 2644154

[22] Cohen BL , Zoëga H , Shah SA , et al. Fatigue is highly associated with poor health‐related quality of life, disability and depression in newly‐diagnosed patients with inflammatory bowel disease, independent of disease activity. Aliment Pharmacol Ther. 2014;39(8):811‐822. 24612278 10.1111/apt.12659 PMC4670472

[23] Bernklev T , Jahnsen J , Aadland E , et al. Health‐related quality of life in patients with inflammatory bowel disease five years after the initial diagnosis. Scand J Gastroenterol. 2004;39(4):365‐373. 15125469 10.1080/00365520310008386

[24] Gregor JC , McDonald JWD , Klar N , et al. An evaluation of utility measurement in Crohn's disease. Inflamm Bowel Dis. 1997;3(4):265‐276. 23282873

[25] Alvestad L , Jelsness‐Jørgensen L‐P , Goll R , et al. Health‐related quality of life in inflammatory bowel disease: a comparison of patients receiving nurse‐led versus conventional follow‐up care. BMC Health Serv Res. 2022;22(1):1602. 36587197 10.1186/s12913-022-08985-1 PMC9805028

[26] Harvey RF , Bradshaw JM . A simple index of Crohn's‐disease activity. Lancet. 1980;315(8167):514. 10.1016/s0140-6736(80)92767-1 6102236

[27] Walmsley RS , Ayres RCS , Pounder RE , Allan RN . A simple clinical colitis activity index. Gut. 1998;43(1):29‐32. 9771402 10.1136/gut.43.1.29 PMC1727189

[28] D'haens G , Sandborn WJ , Feagan BG , et al. A review of activity indices and efficacy end points for clinical trials of medical therapy in adults with ulcerative colitis. Gastroenterology. 2007;132(2):763‐786. 17258735 10.1053/j.gastro.2006.12.038

[29] Bjelland I , Dahl AA , Haug TT , Neckelmann D . The validity of the hospital anxiety and depression scale. J Psychosom Res. 2002;52(2):69‐77. 11832252 10.1016/s0022-3999(01)00296-3

[30] Zigmond AS , Snaith RP . The hospital anxiety and depression scale. Acta Psychiatr Scand. 1983;67(6):361‐370. 6880820 10.1111/j.1600-0447.1983.tb09716.x

[31] Puhan MA , Frey M , Büchi S , Schünemann HJ . The minimal important difference of the hospital anxiety and depression scale in patients with chronic obstructive pulmonary disease. Health Qual Life Outcomes. 2008;6(1):46. 18597689 10.1186/1477-7525-6-46 PMC2459149

[32] Wynne SC , Patel S , Barker RE , et al. Anxiety and depression in bronchiectasis: response to pulmonary rehabilitation and minimal clinically important difference of the hospital anxiety and depression scale. Chron Respir Dis. 2020;17:147997312093329. 10.1177/1479973120933292 PMC7301664 32545998

[33] Keefer L , Kiebles JL , Taft TH . The role of self‐efficacy in inflammatory bowel disease management: preliminary validation of a disease‐specific measure. Inflamm Bowel Dis. 2011;17(2):614‐620. 20848516 10.1002/ibd.21314 PMC3005084

[34] Yoon H , Yang S‐K , So H , et al. Development, validation, and application of a novel tool to measure disease‐related knowledge in patients with inflammatory bowel disease. Korean J Intern Med. 2019;34(1):81‐89. 29172400 10.3904/kjim.2017.104 PMC6325432

[35] Gerner M , Vuillerme N , Aubourg T , et al. Review and analysis of German mobile apps for inflammatory bowel disease management using the mobile application rating scale: systematic search in app stores and content analysis. JMIR Mhealth Uhealth. 2022;10(5):e31102. 35503246 10.2196/31102 PMC9115651

[36] Norouzkhani, N. , Faramarzi, M. , Bahari, A. , Shirvani, J. S. , Eslami, S. , & Tabesh, H. (2024). Inflammatory bowel disease patients’ perspectives of non‐medical needs. BMC Gastroenterology, 24(1). 10.1186/s12876-024-03214-x PMC11016217 38615013

